# Supplementary material for: Baseline Characterization of the Gut Microbiota of Field and Colony Populations of Phlebotomus tobbi and Preliminary Assessment of the Anti-Leishmanial Activity of Cultivable Bacteria
Source: Pathogens. 2026 Jun 23;15(7):658. doi: 10.3390/pathogens15070658 (PMC13416292; doi:10.3390/pathogens15070658)
Supplement: Supplementary file 1 [file pathogens-15-00658-s001.zip › pathogens-4357168-File S1/Taxa2ASV_Decomposer/Family/f__Enterobacteriaceae/abun.plot.top12.pdf]

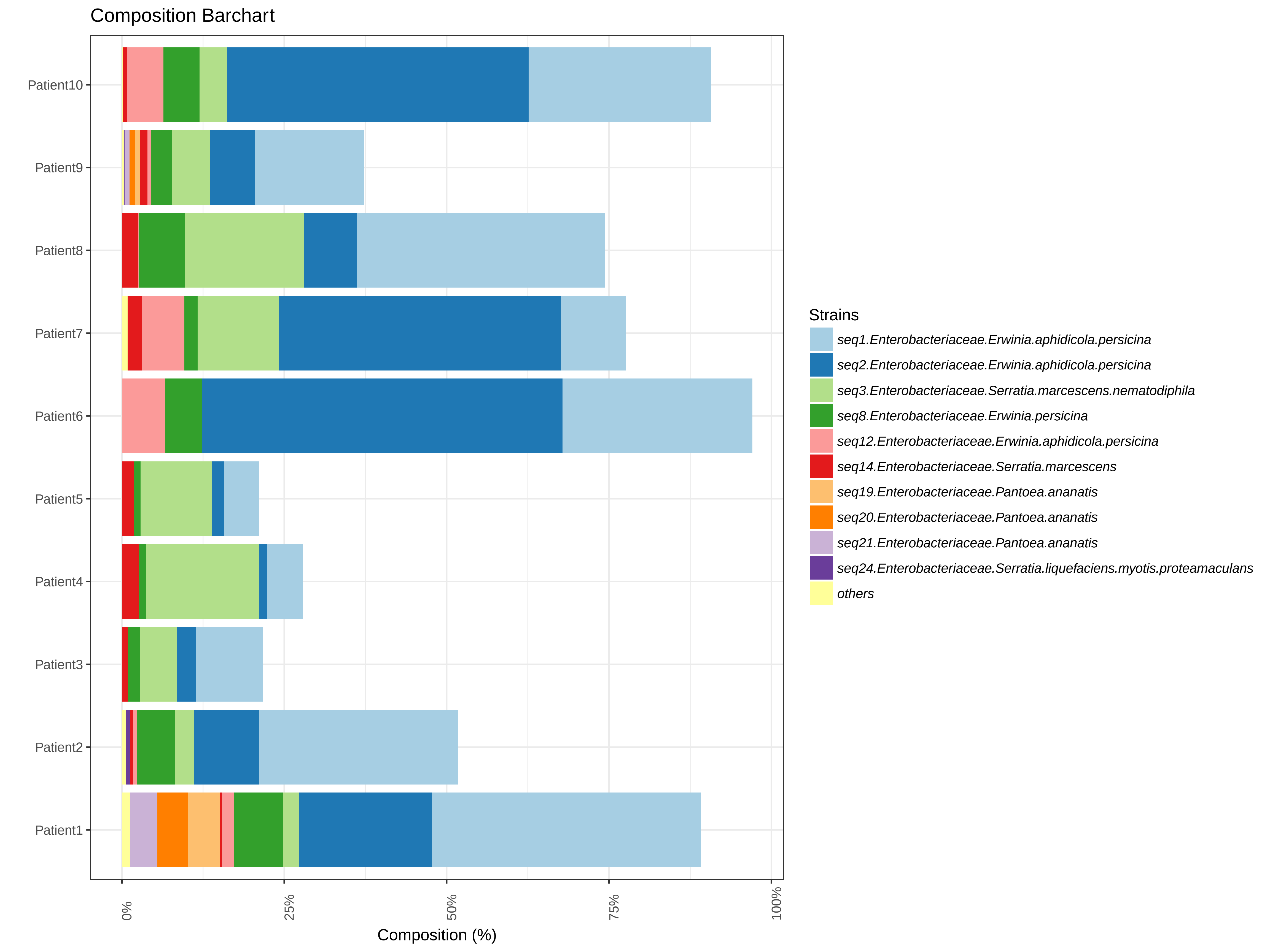

Composition Barchart
Patient10
Patient9
Patient8
Patient7
Patient6
Patient5
Patient4
Patient3
Patient2
Patient1
0%
25%
50%
75%
100%
Composition (%)
Strains
seq1.Enterobacteriaceae.Erwinia.aphidicola.persicina
seq2.Enterobacteriaceae.Erwinia.aphidicola.persicina
seq3.Enterobacteriaceae.Serratia.marcescens.nematodiphila
seq8.Enterobacteriaceae.Erwinia.persicina
seq12.Enterobacteriaceae.Erwinia.aphidicola.persicina
seq14.Enterobacteriaceae.Serratia.marcescens
seq19.Enterobacteriaceae.Pantoea.ananatis
seq20.Enterobacteriaceae.Pantoea.ananatis
seq21.Enterobacteriaceae.Pantoea.ananatis
seq24.Enterobacteriaceae.Serratia.liquefaciens.myotis.proteamaculans
others
